# Poly[[bis­(acetonitrile-κ*N*)bis­[μ_2_-2,2′-(methyl­enedithio)bis­(1,3,4-thia­diazole)-κ^2^ *N* ^4^:*N* ^4′^]copper(II)] bis­(perchlorate) acetonitrile solvate]

**DOI:** 10.1107/S1600536809008708

**Published:** 2009-03-19

**Authors:** Jian-Ge Wang, Jian-Hua Qin, Pu-Zhou Hu

**Affiliations:** a College of Chemistry and Chemical Engineering, Luoyang Normal University, Luoyang 471022, People’s Republic of China

## Abstract

In the title compound, {[Cu(C_5_H_4_N_4_S_4_)_2_(C_2_H_3_N)_2_](ClO_4_)_2_·C_2_H_3_N}_*n*_, the Cu^II^ atom occupies a crystallographic inversion centre and is six-coordinated by six N atoms of four symmetry-related 2,2′-(methyl­enedithio)bis­(1,3,4-thia­diazole) (*L*) ligands and two acetonitrile mol­ecules in a slightly distorted octa­hedral geometry. The ligand *L* adopts an *N*:*N*′-bidentate bridging mode in a *trans* configuration, bridging the Cu atoms *via* translation symmetry, forming a two-dimensional layer-like structure. The perchlorate ions serve as acceptors for inter­molecular C—H⋯O hydrogen bonds, which link the layers into a three-dimensional network. The ClO_4_^−^ anion is disordered with an occupation ratio of 0.658:0.342.

## Related literature

For literature on Cu—N bonds, see: Huang *et al.* (2009 ▶); Qin *et al.* (2009 ▶); Wang *et al.* (2008 ▶).
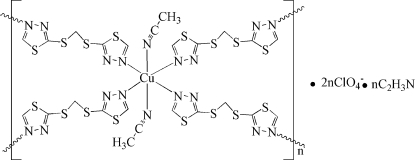


## Experimental

### 

#### Crystal data

[Cu(C_5_H_4_N_4_S_4_)_2_(C_2_H_3_N)_2_](ClO_4_)_2_·C_2_H_3_N
*M* *_r_* = 1764.65
Monoclinic, 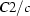

*a* = 19.3144 (18) Å
*b* = 9.9450 (9) Å
*c* = 18.8722 (18) Å
β = 98.876 (1)°
*V* = 3581.6 (6) Å^3^
*Z* = 2
Mo *K*α radiation
μ = 1.28 mm^−1^
*T* = 294 K
0.43 × 0.32 × 0.30 mm

#### Data collection

Bruker SMART CCD area-detector diffractometer
Absorption correction: multi-scan (*SADABS*; Bruker, 1997 ▶) *T*_min_ = 0.608, *T*_max_ = 0.702
12066 measured reflections
3285 independent reflections
2568 reflections with *I* > 2σ(*I*)
*R*_int_ = 0.021

#### Refinement

*R*[*F* ^2^ > 2σ(*F* ^2^)] = 0.057
*wR*(*F* ^2^) = 0.181
*S* = 1.07
3285 reflections
216 parameters
304 restraints
H-atom parameters constrained
Δρ_max_ = 0.86 e Å^−3^
Δρ_min_ = −0.83 e Å^−3^

### 

Data collection: *SMART* (Bruker, 1997 ▶); cell refinement: *SAINT* (Bruker, 1997 ▶); data reduction: *SAINT*; program(s) used to solve structure: *SHELXS97* (Sheldrick, 2008 ▶); program(s) used to refine structure: *SHELXL97* (Sheldrick, 2008 ▶); molecular graphics: *SHELXTL* (Sheldrick, 2008 ▶); software used to prepare material for publication: *SHELXTL*.

## Supplementary Material

Crystal structure: contains datablocks I, global. DOI: 10.1107/S1600536809008708/su2101sup1.cif

Structure factors: contains datablocks I. DOI: 10.1107/S1600536809008708/su2101Isup2.hkl

Additional supplementary materials:  crystallographic information; 3D view; checkCIF report

## Figures and Tables

**Table 1:** Hydrogen-bond geometry (Å, °)

*D*—H⋯*A*	*D*—H	H⋯*A*	*D*⋯*A*	*D*—H⋯*A*
C1—H1⋯O3^i^	0.93	2.35	2.955 (8)	123
C3—H3*A*⋯O1^ii^	0.97	2.41	3.277 (9)	149
C5—H5⋯O1^iii^	0.93	2.45	3.169 (9)	135

Symmetry codes: (i) 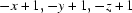; (ii) 
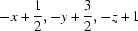
; (iii) 
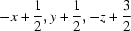
.

**Fig. 1.**
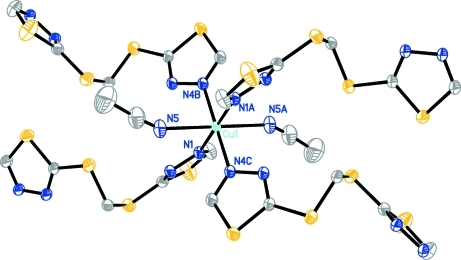
A view of the molecular structure of the cation in the title compound. Displacement ellipsoids are drawn at the 30% probability level. The H atoms, perchlorate ion, and uncoordinated acetonitrile molecules were omitted for clarity [symmetry operations: A: 1/2-x, 3/2-y, -z; B: 1/2-x, -1/2+y, 1/2-z; C: x, 2-y, -1/2+z].

**Fig. 2.**
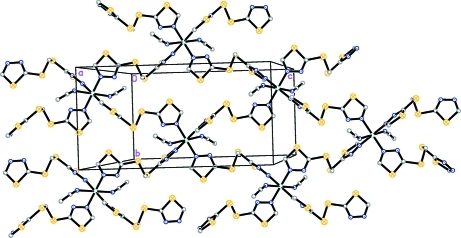
A view of the two-dimensional network in the title compound.

**Fig. 3.**
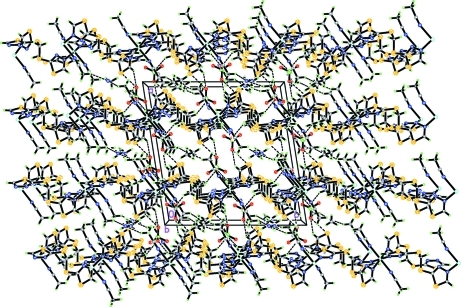
A view down the b axis of the crystal packing of the title compound.

## supplementary crystallographic information

### Comment

The asymmetric unit of the title compound consists of half a Cu^II^ atom, two independent 2,2'-(methylenedithio)bis(1,3,4-thiadiazole) (*L*) ligands, one coordinated acetonitrile molecule, one uncoordinated acetonitrile molecule, and one perchlorate ion (Fig. 1). The Cu^II^ atom is coordinated by six N atoms, from four *L* ligands and two acetonitrile molecules, in a slightly distorted octahedral geometry. All six Cu—N bond distances are within the range expected for such coordination bonds (Huang *et al.*, 2009; Wang *et al.*, 2008; Qin *et al.*, 2009). The ligand *L* adopts a *N:N'*-bidentate bridging mode in *trans* configuration, so bridging the copper atoms *via* translation symmetry to form a two-dimensional layer-like structure, with a bridging Cu···Cu distance of 10.6661 (8) Å (Fig. 2). The centroid-centroid separation and dihedral angle of the thiadiazole rings are 6.3928 (5) Å and 81.86 (13)°, respectively.

In the crystal structure the region between the layes is taken up by perchlorate ions and uncoordinated acetonitrile molecules. The perchlorate ions serve as acceptors for C—H···O hydrogen-bonds, which link the chains into a three-dimensional network (Table 1 and Fig. 3).

### Experimental

The reaction of 2,2'-(methylenedithio)bis(1,3,4-thiadiazole) (0.2 mmol) with Cu(ClO_4_)_2_ (0.1 mmol) in an acetonitrile solution (20 ml) afforded a light blue solid after a few minutes. It was filtered off, washed with acetone, and dried in air. Single crystals, suitable for X-ray analysis, were obtained by slow diffusion of Et_2_O into an acetonitrile solution of the solid.

### Refinement

All H-atoms were positioned geometrically and treated as riding: C—H = 0.93 - 09.7 Å with *U*_iso_(H) = 1.2 or 1.5*U*_eq_(parent C-atom).

### Crystal data

**Table:**

[Cu(C_5_H_4_N_4_S_4_)_2_(C_2_H_3_N)_2_](ClO_4_)_2_·C_2_H_3_N	*F*(000) = 1780
*M**_r_* = 1764.65	*D*_x_ = 1.636 Mg m^−^^3^
Monoclinic, *C*2/*c*	Mo *K*α radiation, λ = 0.71073 Å
Hall symbol: -C 2yc	Cell parameters from 5001 reflections
*a* = 19.3144 (18) Å	θ = 2.3–28.0°
*b* = 9.9450 (9) Å	µ = 1.28 mm^−^^1^
*c* = 18.8722 (18) Å	*T* = 294 K
β = 98.876 (1)°	Block, blue
*V* = 3581.6 (6) Å^3^	0.43 × 0.32 × 0.30 mm
*Z* = 2	

### Data collection

**Table:**

Bruker SMART CCD area-detector diffractometer	3285 independent reflections
Radiation source: fine-focus sealed tube	2568 reflections with *I* > 2σ(*I*)
graphite	*R*_int_ = 0.021
φ and ω scans	θ_max_ = 25.5°, θ_min_ = 2.6°
Absorption correction: multi-scan (*SADABS*; Bruker, 1997)	*h* = −23→23
*T*_min_ = 0.608, *T*_max_ = 0.702	*k* = −12→12
12066 measured reflections	*l* = −22→22

### Refinement

**Table:**

Refinement on *F*^2^	Primary atom site location: structure-invariant direct methods
Least-squares matrix: full	Secondary atom site location: difference Fourier map
*R*[*F*^2^ > 2σ(*F*^2^)] = 0.057	Hydrogen site location: inferred from neighbouring sites
*wR*(*F*^2^) = 0.181	H-atom parameters constrained
*S* = 1.07	*w* = 1/[σ^2^(*F*_o_^2^) + (0.1007*P*)^2^ + 13.6762*P*] where *P* = (*F*_o_^2^ + 2*F*_c_^2^)/3
3285 reflections	(Δ/σ)_max_ < 0.001
216 parameters	Δρ_max_ = 0.86 e Å^−^^3^
304 restraints	Δρ_min_ = −0.83 e Å^−^^3^

### Fractional atomic coordinates and isotropic or equivalent isotropic displacement parameters (Å^2^)

**Table:**

	*x*	*y*	*z*	*U*_iso_*/*U*_eq_	Occ. (<1)
Cl1	0.40159 (11)	0.1887 (3)	0.92446 (12)	0.1040 (7)	0.658 (6)
O1	0.3842 (4)	0.3266 (6)	0.9249 (4)	0.135 (2)	0.658 (6)
O2	0.3626 (4)	0.1399 (7)	0.8620 (4)	0.146 (3)	0.658 (6)
O3	0.4718 (3)	0.1679 (9)	0.9382 (4)	0.139 (2)	0.658 (6)
O4	0.3720 (5)	0.1390 (8)	0.9838 (4)	0.150 (3)	0.658 (6)
Cl1'	0.40159 (11)	0.1887 (3)	0.92446 (12)	0.1040 (7)	0.342 (6)
O1'	0.3333 (3)	0.2118 (9)	0.9325 (5)	0.135 (2)	0.342 (6)
O2'	0.4400 (4)	0.0929 (7)	0.9670 (4)	0.146 (3)	0.342 (6)
O3'	0.4391 (4)	0.3116 (7)	0.9371 (5)	0.139 (2)	0.342 (6)
O4'	0.4083 (5)	0.1678 (8)	0.8518 (3)	0.150 (3)	0.342 (6)
Cu1	0.2500	0.7500	0.0000	0.0403 (3)	
S1	0.41916 (9)	0.9410 (2)	0.16187 (11)	0.0858 (6)	
S2	0.32021 (8)	1.10571 (14)	0.23555 (7)	0.0551 (4)	
S3	0.18788 (8)	0.94899 (14)	0.24653 (6)	0.0542 (4)	
S4	0.17640 (9)	0.85456 (14)	0.39769 (7)	0.0584 (4)	
N1	0.3156 (2)	0.8589 (4)	0.07448 (19)	0.0424 (9)	
N2	0.2872 (2)	0.9444 (4)	0.12019 (19)	0.0421 (9)	
N3	0.2193 (2)	1.0883 (4)	0.36936 (19)	0.0445 (9)	
N4	0.2208 (2)	1.0822 (4)	0.44266 (19)	0.0425 (9)	
N5	0.1534 (3)	0.7792 (6)	0.0641 (3)	0.0662 (13)	
C1	0.3821 (3)	0.8491 (7)	0.0899 (3)	0.0651 (16)	
H1	0.4084	0.7955	0.0637	0.078*	
C2	0.3356 (3)	0.9939 (5)	0.1685 (2)	0.0454 (11)	
C3	0.2265 (3)	1.1032 (5)	0.2201 (3)	0.0498 (12)	
H3A	0.2105	1.1181	0.1694	0.060*	
H3B	0.2095	1.1773	0.2461	0.060*	
C4	0.1974 (3)	0.9770 (5)	0.3392 (2)	0.0410 (10)	
C5	0.1996 (3)	0.9686 (5)	0.4642 (3)	0.0509 (13)	
H5	0.1973	0.9505	0.5121	0.061*	
C6	0.1050 (4)	0.7464 (9)	0.0852 (4)	0.088 (2)	
C7	0.0400 (6)	0.6925 (15)	0.1112 (7)	0.144 (4)	
H7A	−0.0006	0.7375	0.0865	0.216*	
H7B	0.0434	0.7083	0.1618	0.216*	
H7C	0.0361	0.5977	0.1019	0.216*	
N6	1.000 (2)	−0.171 (2)	0.2314 (17)	0.212 (10)	0.50
C8	0.9970 (14)	−0.059 (2)	0.2284 (11)	0.208 (11)	0.50
C9	0.993 (2)	0.093 (2)	0.2242 (18)	0.204 (11)	0.50
H9A	1.0400	0.1296	0.2300	0.306*	0.50
H9B	0.9696	0.1270	0.2617	0.306*	0.50
H9C	0.9682	0.1199	0.1785	0.306*	0.50

### Atomic displacement parameters (Å^2^)

**Table:**

	*U*^11^	*U*^22^	*U*^33^	*U*^12^	*U*^13^	*U*^23^
Cl1	0.0787 (12)	0.1298 (17)	0.0987 (14)	0.0298 (12)	−0.0016 (10)	−0.0166 (13)
O1	0.118 (4)	0.147 (4)	0.137 (4)	0.040 (4)	0.012 (4)	−0.018 (4)
O2	0.136 (5)	0.150 (4)	0.141 (4)	0.019 (4)	−0.016 (4)	−0.013 (4)
O3	0.105 (4)	0.156 (4)	0.156 (5)	0.017 (4)	0.018 (4)	−0.001 (4)
O4	0.141 (5)	0.173 (5)	0.138 (4)	0.009 (4)	0.036 (4)	0.000 (4)
Cl1'	0.0787 (12)	0.1298 (17)	0.0987 (14)	0.0298 (12)	−0.0016 (10)	−0.0166 (13)
O1'	0.118 (4)	0.147 (4)	0.137 (4)	0.040 (4)	0.012 (4)	−0.018 (4)
O2'	0.136 (5)	0.150 (4)	0.141 (4)	0.019 (4)	−0.016 (4)	−0.013 (4)
O3'	0.105 (4)	0.156 (4)	0.156 (5)	0.017 (4)	0.018 (4)	−0.001 (4)
O4'	0.141 (5)	0.173 (5)	0.138 (4)	0.009 (4)	0.036 (4)	0.000 (4)
Cu1	0.0650 (6)	0.0348 (4)	0.0214 (4)	0.0057 (3)	0.0075 (3)	−0.0001 (3)
S1	0.0569 (9)	0.1161 (16)	0.0808 (12)	0.0020 (9)	−0.0006 (8)	−0.0383 (11)
S2	0.0796 (9)	0.0467 (7)	0.0378 (7)	−0.0078 (6)	0.0053 (6)	−0.0117 (5)
S3	0.0840 (10)	0.0491 (7)	0.0300 (6)	−0.0130 (6)	0.0106 (6)	−0.0096 (5)
S4	0.0932 (11)	0.0443 (7)	0.0401 (7)	−0.0216 (7)	0.0176 (7)	−0.0046 (5)
N1	0.058 (3)	0.042 (2)	0.0291 (18)	0.0056 (18)	0.0118 (17)	0.0004 (16)
N2	0.058 (2)	0.039 (2)	0.0292 (18)	0.0026 (18)	0.0076 (17)	−0.0023 (15)
N3	0.067 (3)	0.041 (2)	0.0275 (18)	−0.0069 (19)	0.0127 (17)	−0.0023 (16)
N4	0.064 (3)	0.040 (2)	0.0253 (18)	−0.0051 (18)	0.0104 (17)	−0.0005 (15)
N5	0.072 (3)	0.082 (3)	0.049 (3)	0.001 (3)	0.022 (2)	−0.006 (2)
C1	0.061 (4)	0.078 (4)	0.057 (3)	0.007 (3)	0.011 (3)	−0.020 (3)
C2	0.063 (3)	0.040 (2)	0.033 (2)	−0.002 (2)	0.007 (2)	0.0015 (19)
C3	0.085 (4)	0.037 (2)	0.029 (2)	0.006 (2)	0.013 (2)	−0.0002 (19)
C4	0.054 (3)	0.041 (2)	0.029 (2)	−0.003 (2)	0.0103 (19)	−0.0025 (19)
C5	0.078 (4)	0.046 (3)	0.030 (2)	−0.011 (3)	0.013 (2)	−0.004 (2)
C6	0.085 (4)	0.112 (5)	0.066 (4)	−0.001 (4)	0.012 (3)	−0.008 (3)
C7	0.116 (6)	0.184 (8)	0.138 (7)	−0.028 (6)	0.037 (5)	0.012 (7)
N6	0.208 (12)	0.212 (11)	0.217 (14)	−0.002 (10)	0.033 (10)	0.002 (9)
C8	0.202 (12)	0.209 (12)	0.213 (14)	0.002 (9)	0.030 (9)	0.003 (9)
C9	0.197 (14)	0.205 (12)	0.211 (15)	0.003 (10)	0.036 (10)	0.002 (9)

### Geometric parameters (Å, °)

**Table:**

Cl1—O3	1.356 (5)	N1—C1	1.277 (8)
Cl1—O2	1.385 (5)	N1—N2	1.382 (5)
Cl1—O1	1.411 (5)	N2—C2	1.298 (6)
Cl1—O4	1.422 (5)	N3—C4	1.286 (6)
Cl1'—O1'	1.371 (5)	N3—N4	1.380 (5)
Cl1'—O2'	1.385 (5)	N4—C5	1.288 (6)
Cl1'—O4'	1.413 (5)	N4—Cu1^iv^	2.021 (4)
Cl1'—O3'	1.422 (5)	N5—C6	1.119 (8)
Cu1—N4^i^	2.021 (4)	C1—H1	0.9300
Cu1—N4^ii^	2.021 (4)	C3—H3A	0.9700
Cu1—N1^iii^	2.050 (4)	C3—H3B	0.9700
Cu1—N1	2.050 (4)	C5—H5	0.9300
Cu1—N5^iii^	2.393 (5)	C6—C7	1.515 (11)
Cu1—N5	2.393 (5)	C7—H7A	0.9600
S1—C1	1.700 (6)	C7—H7B	0.9600
S1—C2	1.720 (6)	C7—H7C	0.9600
S2—C2	1.744 (5)	N6—C8	1.114 (6)
S2—C3	1.789 (6)	C8—C9	1.523 (8)
S3—C4	1.752 (4)	C9—H9A	0.9600
S3—C3	1.809 (5)	C9—H9B	0.9600
S4—C5	1.699 (5)	C9—H9C	0.9600
S4—C4	1.734 (5)		
			
O3—Cl1—O2	120.3	N2—N1—Cu1	119.4 (3)
O3—Cl1—O1	112.3	C2—N2—N1	111.1 (4)
O2—Cl1—O1	104.3	C4—N3—N4	111.1 (4)
O3—Cl1—O4	107.9	C5—N4—N3	113.3 (4)
O2—Cl1—O4	108.5	C5—N4—Cu1^iv^	129.1 (3)
O1—Cl1—O4	102.0	N3—N4—Cu1^iv^	117.5 (3)
O1'—Cl1'—O2'	119.3	C6—N5—Cu1	154.6 (6)
O1'—Cl1'—O4'	111.4	N1—C1—S1	115.2 (4)
O2'—Cl1'—O4'	109.9	N1—C1—H1	122.4
O1'—Cl1'—O3'	108.2	S1—C1—H1	122.4
O2'—Cl1'—O3'	106.3	N2—C2—S1	114.5 (4)
O4'—Cl1'—O3'	99.7	N2—C2—S2	124.6 (4)
N4^i^—Cu1—N4^ii^	180.0	S1—C2—S2	121.0 (3)
N4^i^—Cu1—N1^iii^	91.30 (15)	S2—C3—S3	114.6 (3)
N4^ii^—Cu1—N1^iii^	88.70 (15)	S2—C3—H3A	108.6
N4^i^—Cu1—N1	88.70 (15)	S3—C3—H3A	108.6
N4^ii^—Cu1—N1	91.30 (15)	S2—C3—H3B	108.6
N1^iii^—Cu1—N1	180.0	S3—C3—H3B	108.6
N4^i^—Cu1—N5^iii^	89.75 (18)	H3A—C3—H3B	107.6
N4^ii^—Cu1—N5^iii^	90.25 (18)	N3—C4—S4	114.6 (3)
N1^iii^—Cu1—N5^iii^	92.10 (17)	N3—C4—S3	123.8 (3)
N1—Cu1—N5^iii^	87.90 (17)	S4—C4—S3	121.7 (3)
N4^i^—Cu1—N5	90.25 (18)	N4—C5—S4	114.4 (4)
N4^ii^—Cu1—N5	89.75 (18)	N4—C5—H5	122.8
N1^iii^—Cu1—N5	87.90 (17)	S4—C5—H5	122.8
N1—Cu1—N5	92.10 (17)	N5—C6—C7	176.0 (10)
N5^iii^—Cu1—N5	180.0	C6—C7—H7A	109.5
C1—S1—C2	86.5 (3)	C6—C7—H7B	109.5
C2—S2—C3	98.9 (2)	H7A—C7—H7B	109.5
C4—S3—C3	99.0 (2)	C6—C7—H7C	109.5
C5—S4—C4	86.6 (2)	H7A—C7—H7C	109.5
C1—N1—N2	112.7 (4)	H7B—C7—H7C	109.5
C1—N1—Cu1	127.6 (4)	N6—C8—C9	180.0
			
N4^i^—Cu1—N1—C1	63.0 (5)	C2—S1—C1—N1	−0.1 (5)
N4^ii^—Cu1—N1—C1	−117.0 (5)	N1—N2—C2—S1	0.3 (5)
N5^iii^—Cu1—N1—C1	−26.8 (5)	N1—N2—C2—S2	179.9 (3)
N5—Cu1—N1—C1	153.2 (5)	C1—S1—C2—N2	−0.1 (4)
N4^i^—Cu1—N1—N2	−109.4 (3)	C1—S1—C2—S2	−179.8 (4)
N4^ii^—Cu1—N1—N2	70.6 (3)	C3—S2—C2—N2	6.9 (5)
N5^iii^—Cu1—N1—N2	160.8 (3)	C3—S2—C2—S1	−173.5 (3)
N5—Cu1—N1—N2	−19.2 (3)	C2—S2—C3—S3	71.7 (3)
C1—N1—N2—C2	−0.4 (6)	C4—S3—C3—S2	78.6 (3)
Cu1—N1—N2—C2	173.1 (3)	N4—N3—C4—S4	0.2 (5)
C4—N3—N4—C5	−0.6 (6)	N4—N3—C4—S3	179.3 (4)
C4—N3—N4—Cu1^iv^	−177.6 (3)	C5—S4—C4—N3	0.2 (4)
N4^i^—Cu1—N5—C6	−53.6 (14)	C5—S4—C4—S3	−179.0 (4)
N4^ii^—Cu1—N5—C6	126.4 (14)	C3—S3—C4—N3	6.7 (5)
N1^iii^—Cu1—N5—C6	37.7 (14)	C3—S3—C4—S4	−174.2 (3)
N1—Cu1—N5—C6	−142.3 (14)	N3—N4—C5—S4	0.8 (6)
N2—N1—C1—S1	0.3 (7)	Cu1^iv^—N4—C5—S4	177.3 (3)
Cu1—N1—C1—S1	−172.5 (3)	C4—S4—C5—N4	−0.6 (5)

Symmetry codes: (i) −*x*+1/2, *y*−1/2, −*z*+1/2; (ii) *x*, −*y*+2, *z*−1/2; (iii) −*x*+1/2, −*y*+3/2, −*z*; (iv) −*x*+1/2, *y*+1/2, −*z*+1/2.

### Hydrogen-bond geometry (Å, °)

**Table:**

*D*—H···*A*	*D*—H	H···*A*	*D*···*A*	*D*—H···*A*
C1—H1···O3^v^	0.93	2.35	2.955 (8)	123
C3—H3A···O1^vi^	0.97	2.41	3.277 (9)	149
C5—H5···O1^vii^	0.93	2.45	3.169 (9)	135

Symmetry codes: (v) −*x*+1, −*y*+1, −*z*+1; (vi) −*x*+1/2, −*y*+3/2, −*z*+1; (vii) −*x*+1/2, *y*+1/2, −*z*+3/2.

## References

[bb1] Bruker (1997). *SMART*, *SAINT* and *SADABS* Bruker AXS Inc., Madison, Wisconsin, USA.

[bb2] Huang, H.-M., Ju, F.-Y., Wang, J.-G. & Qin, J.-H. (2009). *Acta Cryst.* E**65**, m80–m81. 10.1107/S1600536808041202 PMC2967916 21581546

[bb3] Qin, J.-H., Wang, J.-G. & Hu, P.-Z. (2009). *Acta Cryst.* E**65**, m349–m350. 10.1107/S1600536809006722 PMC2968698 21582113

[bb4] Sheldrick, G. M. (2008). *Acta Cryst.* A**64**, 112–122. 10.1107/S0108767307043930 18156677

[bb5] Wang, J. G., Qin, J. H., Hu, P. Z. & Zhao, B. T. (2008). *Z. Kristallogr. New Cryst. Struct.* **223**, 225–227.

